# Outside the Thorax: Doege–Potter Syndrome Presenting as a Retroperitoneal Abdominal Mass

**DOI:** 10.1155/2021/9919321

**Published:** 2021-05-19

**Authors:** Daniel M. Berrebi, Oksana Symczyk, Taylor Cater, Ayodele Adelanwa, Patrick Bacaj, Alan A. Thomay, Adnan Haider

**Affiliations:** ^1^School of Medicine, West Virginia University, Morgantown, WV, USA; ^2^Department of Endocrinology, Department of Medicine, West Virginia University, Morgantown, WV, USA; ^3^Department of Pathology, Anatomy and Laboratory Medicine, West Virginia University, Morgantown, WV, USA; ^4^Department of Surgery, Division of Surgical Oncology, West Virginia University, Morgantown, WV, USA

## Abstract

**Objective:**

We present a case of refractory hypoglycemia, weight loss, and retroperitoneal solitary fibrous tumor. *Case report*. A 68-year-old female presented with symptomatic hypoglycemia, weight loss, and abdominal mass identified on CT scan of the abdomen. Blood work during symptomatic hypoglycemia was consistent with an IGF-2-producing tumor. The abdominal mass pathology was consistent with solitary fibrous tumor surrounding the adrenal gland, and resection resulted in complete resolution of hypoglycemia. *Discussion*. Understanding the biochemical mechanisms behind glucose regulation is necessary to diagnose and adequately treat Doege–Potter syndrome, a paraneoplastic syndrome observed in patients with solitary fibrous tumors. Solitary fibrous tumors can be characterized by specific histologic and immunohistochemical studies.

**Conclusion:**

This report describes the clinical workup of a patient presenting with hypoglycemia and a retroperitoneal tumor. This case is unique because of its presentation with severe, refractory hypoglycemia and the tumor's location in the retroperitoneum, given the majority of solitary fibrous tumors are found in the lungs originating from the pleura.

## 1. Case Report

**Figure 1:**
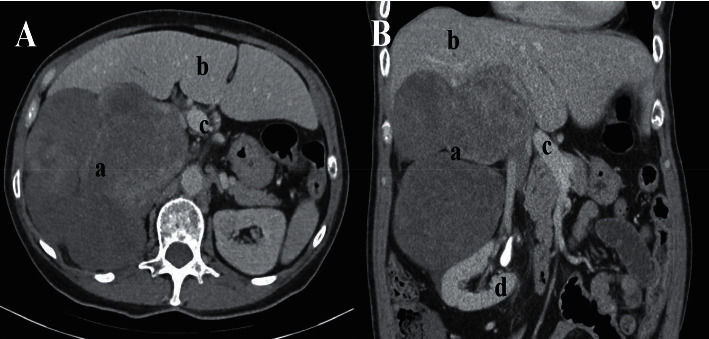
CT Scan of retroperitoneal mass. (a) Axial slice of contrast enhanced CT of the abdomen demonstrating a large right retroperitoneal tumor (A) with mass effect upon the right lobe of the liver (B) and displacement of the vena cava and porta hepatis (C) medially. (b) Coronal slice of contrast enhanced CT of the abdomen again showing the tumor, with displacement of the right kidney (D) inferiorly.

A 68-year-old female patient presented to the emergency department with refractory neuroglycopenic symptoms. In the emergency room, her glucose level was noted to be <50 mg/dL on multiple occasions despite repeated administrations of IV dextrose and PO intake. She described a 3-week history of episodes characterized by confusion, diaphoresis, and severe weakness which were improved with ingestion of food. She also described a weight loss of 30 pounds over the last 4-5 months despite normal appetite. She had no history of type 1 or type 2 diabetes mellitus and did not use insulin or antidiabetic medications. Medical history included hypertension and gastroesophageal reflux, and she did not endorse a history of smoking, alcohol, or recreational drug use. No pertinent family history was identified. On physical examination, she was found to have a palpable, mildly tender right upper quadrant mass and palpable hepatomegaly. A CT scan demonstrated evidence of an 18.6 cm mass projecting inferior to the liver and superior to the right kidney, in the approximate location of the right adrenal gland (Figure 1). The mass was described as lobular and formed by 3 confluent mass lesions. No other suspicious lesions were described in the report. In addition, venogram showed severe extrinsic compression of the inferior vena cava secondary to the retroperitoneal mass.

**Figure 2:**
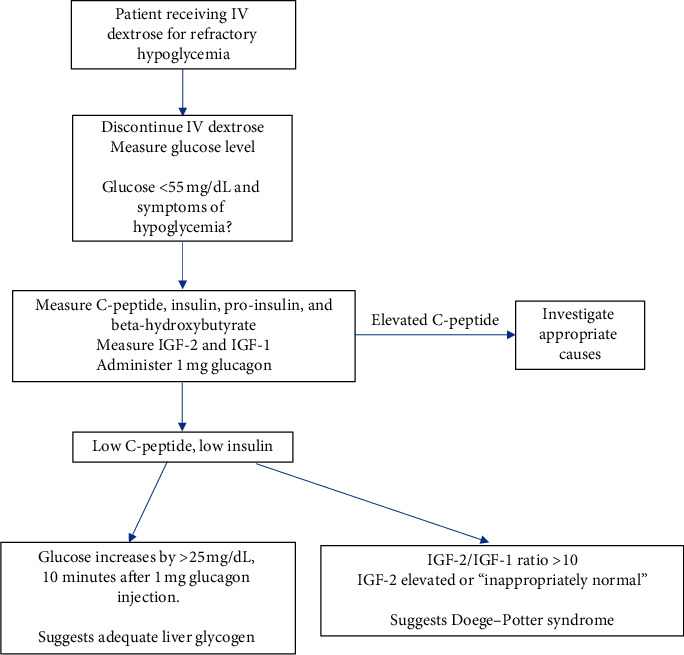
Clinical workup of Doege–Potter syndrome.

Clinical workup of her hypoglycemia proceeded as follows (Figure 2). Hypoglycemia was initially prevented with a maintenance dextrose drip; however, this was discontinued to allow clinical observation of hypoglycemic episodes ideally proving or disproving Whipple's triad. Whipple's triad includes a venous glucose level less than 55 mg/dL, symptoms of hypoglycemia, and resolution of symptoms after glucose administration. Dextrose drip was discontinued, her glucose level decreased to 47 mg/dL, and she became symptomatic. At that time, C-peptide, insulin, pro-insulin, and beta-hydroxybutyrate levels were measured and all were found to be low, suggesting IGF-2 secretion. IGF-2 was measured during that hypoglycemic episode but resulted within the normal range. This is classified as inappropriately normal because IGF-2 levels are expected to be suppressed in the setting of hypoglycemia (Table 1). The patient was given a 1 mg dose of glucagon, and her glucose level increased to 82 mg/dL with resolution of symptoms. Increase in glucose value of greater then 25 mg/dl represent adequate glycogen stores in the liver, yet repeated and persistent hypoglycemia infers that the patient's hepatocytes were not secreting glucose despite severe hypoglycemia. Dextrose drip was resumed to maintain safe glucose levels.

Screening for use of oral hypoglycemic agents to rule out factitious hypoglycemia was negative. Antibodies to insulin were also undetectable. Our surgical colleagues recommended ruling out adrenal hypersecretion, as elevated precursor hormones are often seen in rapidly dividing adrenocortical carcinoma. Serum renin, aldosterone, 11-deoxycorticosterone, and 17-hydroxyprogesterone were all within normal limits. 5-HIAA and urine metanephrine levels were normal as well, which lessened suspicion for pheochromocytoma or neuroendocrine tumor. Although these conditions are not classically associated with hypoglycemia, we felt it would be useful to exclude additional causes of a large retroperitoneal mass.

**Figure 3:**
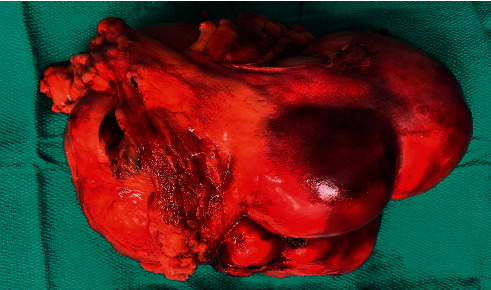
Gross image of solitary fibrous tumor. Specimen weighed 2270 grams with dimensions of 22.0 cm × 18.0 cm × 12.0 cm. Solitary fibrous tumors often appear as a well-circumscribed mass with tan-white homogeneous cut surfaces that can be cystic or hemorrhagic.

Given the in-depth negative workup other than the abdominal mass, the decision was made to pursue resection. The gross pathology specimen (Figure 3) showed a mass weighing 2270 gm. Following removal, the patient's hypoglycemia resolved and 2 days later, IGF-2 was re-measured and found to be significantly lower than that measured preoperatively. During recovery, she had episodes of hyperglycemia, which could be explained by the sudden decrease in IGF-2 levels triggering a new equilibrium for glucose homeostasis.

Unfortunately, IGF-1 level, which was drawn at the time of a hypoglycemic episode, could not be resulted because the sample was hemolyzed en route to the laboratory. By the time we were alerted, the patient was in pre-op for surgical resection. It would have been ideal to have this value, but this case highlights that clinical suspicion in the setting of appropriate imaging, low insulin, C-peptide, and beta-hydroxybutyrate, and an increase in glucose following glucagon injection point to an IGF-2-producing tumor. Finally, in the setting of an acutely ill patient, we anticipate a low/suppressed IGF-1 level.

A follow-up CT scan one month after surgery demonstrated interval resection of the previously noted large, lobulated right suprarenal mass. There were no findings to suggest residual or recurrent disease.

## 2. Discussion

Solitary fibrous tumors (SFTs) arise from mesenchymal cells and account for less than 2% of all soft tissue tumors [1]. SFTs usually manifest as slowly growing, asymptomatic masses and are benign in clinical course. Cases of aggressive SFTs with local invasion have been reported [2]. SFTs typically originate within the thorax but can arise anywhere, and to date, there have been fewer than 100 cases reported of retroperitoneal SFTs. Rarely, SFTs can present with paraneoplastic syndromes such as hypoglycemia, specifically referred to as the Doege–Potter syndrome, which was first reported in the 1930s [3, 4].

The effectiveness of the normal physiological reactions to falling blood glucose concentrations makes hypoglycemia an uncommon clinical event. The first response to falling serum glucose concentration is a decrease in the secretion of endogenous insulin. Once glucose concentrations fall below the normal physiological range, counter-regulatory hormones are released in response simulating glucagon, cortisol, catecholamine, and growth hormone secretion [5]. Together these effects stimulate hepatic glucose production and decrease glucose utilization in peripheral tissues. In the event of recurrent episodes of hypoglycemia, the glycemic threshold of physiological defenses shifts to a lower glucose concentration making diagnosis and treatment critical to prevent fatal consequences as a result of hypoglycemic unawareness [6].

Although hypoglycemia from non-islet cell tumors is very rare, when present it often results from overproduction of pro-insulin-like growth factor-2 (IGF-2) which is an incompletely processed high molecular weight protein. Due to its poor affinity to serum IGF-2 binding proteins, IGF-2 freely enters tissue eliciting its insulin-like effects and therefore leads to hypoglycemia [7, 8]. When glucose levels fall during a fasting state, compensatory mechanisms result in a rise in ketones such as *β*-hydroxybutyrate, as an alternative energy source for the brain [9, 10]. However, this rise in ketones is blunted by hypoglycemia stemming from IGF-2-producing tumors [11]. Just like insulin, IGF-2 stimulates uptake of glucose by skeletal muscles [12]. Continued stimulation of insulin receptors by IGF-2 leads to further suppression of free fatty acid release as well as inhibition of gluconeogenesis, glycogenolysis, and ketogenesis by the liver [11, 12]. Suppression of growth hormone and IGF-1 also results due to elevated IGF-2 levels. These effects in turn lead to an elevated IGF-2 to IGF-1 ratio. An IGF-2 to IGF-1 ratio of greater than 10 is typically revealing of non-islet cell tumor hypoglycemia (NICTH) [13]. Although the IGF-2 level was found to be normal in this case, IGF-2-producing NICTH has been previously reported with both normal and elevated IGF-2 levels [14]. We would classify the IGF-2 level as inappropriately normal in the setting of significant hypoglycemia in such cases.

**Figure 4:**
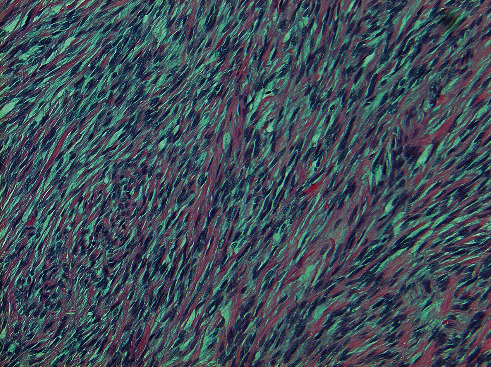
Histologic appearance of solitary fibrous tumor. Photomicrograph of solitary fibrous tumor at 100x magnification demonstrating the classic pattern-less pattern of uniform, spindled to ovoid fibroblastic cells.

From a pathology perspective, the gross appearance of a typical or conventional SFT is a well-circumscribed mass with tan-white homogeneous cut surfaces that can be cystic or hemorrhagic with sizes ranging from 1 cm to 20 cm (Figure 3). Histologically, SFTs are often described as having a “pattern-less pattern” (Figure 4) which refers to a storiform arrangement of neoplastic spindle cells in a stroma with variable amounts of collagen and large thin-walled branching “staghorn” appearing vessels and medium-sized vessels. A diagnosis of malignant SFT is rendered when the tumor displays high cellularity, high mitotic activity (more than 4 mitoses per 10HPF), cytological pleomorphism, and necrosis on histopathologic evaluation [15].

Immunohistochemically, CD34, Bcl-2, and CD99 SFT usually show a strong diffuse pattern of staining; however, in 5–10% of SFT, CD34 can be negative. These markers can also show positivity in other neoplasms of mesenchymal differentiation, limiting the utility of these markers, particularly CD34, in the workup of the differential diagnosis [16]. The most sensitive and specific marker for the diagnosis of both conventional and malignant SFTs is the detection of NAB2-STAT6 fusion by next-generation sequencing [17]. Immunohistochemical expression of the STAT6 protein in tumor cell nuclei is a sensitive and specific marker for SFT. Cytoplasmic expression of STAT6 can sometimes be seen in other neoplasms such as synovial sarcoma, well-differentiated and dedifferentiated liposarcomas, and desmoid fibromatosis. The distinguishing feature for SFTs lies in the pattern of expression. SFT exclusively shows a nuclear pattern of STAT6 expression, whereas these other neoplasms display a nuclear and cytoplasmic pattern of staining [18].

The only definitive treatment for NICTH is complete tumor resection. As in previously reported cases, our patient's symptoms completely resolved once the tumor was resected. Normalization of plasma glucose levels occurred shortly after tumor resection. Postoperatively, during the recovery phase, she experienced episodes of postprandial hyperglycemia. This was presumably secondary to residually suppressed insulin secretion from pancreatic *β* cells. This resolved with decreasing levels of IGF-2 and a re-equilibration in glucose homeostasis. A study by Le Jeune et al. [19] showed that the recurrence-free survival generally exceeds 90% [19].

In cases of incomplete resection or tumors that cannot be surgically removed, there are medical management options. Han et al. [20] describes the efficacy of various glucocorticoid regimens, which suppress production of IGF-2 and have been shown to improve hypoglycemia in a dose-dependent manner. In combination with steroids, some providers initiate growth hormone therapy, which may have a synergistic effect. In a patient with Doege–Potter syndrome and end-stage renal disease, Shekhar et al. [21] described use of dexamethasone and recombinant growth hormone to manage hypoglycemia; however, the patient became increasingly dependent on IV dextrose and eventually an attempt to de-bulk the tumor was made. Chemotherapy and radiation can be considered, but according to Han et al., IGF-2-producing solitary fibrous tumors often respond poorly to these interventions.

## 3. Conclusion

SFTs have been described in extrapleural sites such as the upper respiratory tract and the orbits but are most commonly found in the lungs originating from the pleura. If hypoglycemia is demonstrated in connection with a solitary fibrous tumor, it is referred to as the Doege–Potter syndrome. This case is unique because of its presentation with severe hypoglycemia and its location “outside the thoracic cavity.”

## Figures and Tables

**Table 1:** Relevant laboratory values and corresponding reference ranges.

Test	Result	Range
Plasma glucose after dextrose discontinuation	47 mg/dL	
C-peptide	0.1 ng/mL	0.9–7.1 ng/mL
Beta-hydroxybutyrate	0.10 mmol/L	<0.40 mmol/L
Pro-insulin	2.8 pmol/L	3.6–22 pmol/L
Insulin	<2.0 mIU/mL	2.5–24.0 mIU/mL
Insulin-like growth factor-2	382 ng/mL	333–967 ng/mL
Insulin antibodies	Negative	

## Abbreviations

DPS: Doege–Potter syndrome
IGF-2: Insulin-like growth factor-2
SFT: Solitary fibrous tumor
NICTH: Non-islet cell tumor hypoglycemia.
